# Evaluation of Point-of-Care Ultrasound Use in Emergency Medicine Residents: An Observational Study

**DOI:** 10.5811/westjem.21200

**Published:** 2025-05-19

**Authors:** Michael Fareri, Matthew VandeHei, Benjamin Schnapp, Corlin Jewell, Michael R. Lasarev, Roxana Alexandridis, Dana Resop, Sara Damewood, Hani I. Kuttab

**Affiliations:** * University of Wisconsin-Madison, Department of Emergency Medicine, Madison, Wisconsin; † University of Wisconsin-Madison, Department of Biostatistics and Medical Informatics, Madison, Wisconsin

## Abstract

**Introduction:**

Point-of-care ultrasound (POCUS) is integral to emergency medicine (EM) training. It is unclear how EM residents use POCUS and how these skills are maintained as they progress in residency training. The purpose of this study was to evaluate resident use of POCUS at various timepoints in EM training.

**Methods:**

This was a retrospective cohort study of EM residents at a single, three-year training program between July 1, 2014–June 30, 2022. Residents were included if they had completed three consecutive years of training and an ultrasound rotation in their postgraduate year (PGY)-1. The following time points were assessed: PGY-1 rotation and 3-, 6-, 12-, 18-, and 24-months post-rotation. Number of scans, accuracy of interpretation, acceptability for credit, and percentage of technically limited studies (TLS) were collected at each point. We analyzed performance characteristics using mixed-effects binomial logistic regression with time as a fixed effect and resident as a random effect. Models were fit separately for each performance characteristic and likelihood ratio tests were performed to determine whether performance varied over time.

**Results:**

A total of 65 residents were included with a total of 13,229 exams performed during the study period. Cardiac and focused assessment with sonography in trauma examinations were performed most commonly. Overall accuracy of all exams during the examination period was 97.1% (95% confidence interval [CI] 96.2–98.0%), TLS was 14.5% (95% CI 9.7–20.6%), and acceptability was 82.9% (95% CI 76.3–88.2%). Trend over time (3, 6, 12, 18, and 24 months) found no differences in accuracy (*P* = 0.84), TLS (*P* = 0.20), or acceptability (*P* = 0.28). Further analyses by individual exam types also showed no significant differences in accuracy, acceptability, nor TLS.

**Conclusion:**

Accuracy, acceptability, and percentage of technically limited scans did not significantly vary over time, suggesting that POCUS skills are maintained from PGY-1 rotation to each time point evaluated in this study.

## INTRODUCTION

The Accreditation Council for Graduate Medical Education lists point-of-care ultrasound (POCUS) as a core competency for emergency medicine (EM) residency training.^1^ The American College of Emergency Physicians (ACEP) provides guidance on what this training should entail, including a two-week rotation as a postgraduate year (PGY)-1 resident and at least one additional week in subsequent years of training.^2^,^3^ As a result, it is standard to have a dedicated POCUS rotation in EM training; however, training curriculums vary in length and timing.^4^ Additionally, there are often limitations of institutional curriculums and exposure to pathology based on one’s training setting.^5^

Some data suggests a decrease in POCUS use and skill as the time from initial training progresses.^6^–^9^ A study of a four-year EM residency demonstrated fewer scans performed between the PGY-1 to PGY-2 year, as well as between the PGY-3 and PGY-4 year.^9^ In this same group, it was observed that the rate of technically limited studies (TLS, ie, lacking the proper number of views, or the images themselves were not interpretable) increased as training progressed.^9^ Training users in POCUS is resource-intensive, often requiring an asynchronous curriculum, dedicated one-on-one time at the bedside with faculty, and image review for feedback. Thus, there is a need to better understand the use, accuracy, and retention of POCUS skills among EM residents as they progress through training.

Our primary objective in this study was to evaluate use of POCUS among EM residents at various time points through residency training. The secondary objective was to evaluate the trends in accuracy, acceptability, and percentage of TLS at specific time points as potential targets for re-education.

## METHODS

### Study Design

This was a retrospective, observational cohort study of a three-year EM residency training program at a single, academic medical center from July 1, 2014–June 30, 2022, including six residency classes. Data prior to 2014 was not available for inclusion. This study was reviewed by the institutional review board and determined to be exempt from review.

### Study Protocol

Dates of the PGY-1 ultrasound rotation were obtained from information stored in an online residency management program (MedHub, Minneapolis, MN) and confirmed with the residency education coordinator. All POCUS images are stored in a cloud-based storage software program (QPathE, Telexy Healthcare Inc, British Columbia, Canada). Total number of scans obtained, accuracy of interpretation, acceptability for credentialing, and percentage of TLS were collected using the analytics function in QPathE.

We collected data at five time points: PGY-1 rotation (defined as the ultrasound rotation start date to rotation end date); three months (end date of PGY-1 rotation plus 90 days); six months (end date of PGY-1 rotation plus 180 days), 12 months (end date of PGY-1 rotation plus 360 days), 18 months (end date of PGY-1 rotation plus 540 days); and 24 months (end date of PGY-1 rotation plus 720 days). Outcome measures were a cumulative total of all scans performed to that specific time point. These time points were chosen a priori based on consensus agreement among faculty members from the residency leadership team and members of the division of ultrasound as time points of interest for potential re-education. Approximately 700 studies total (~4.8%) were performed prior to the PGY-1 rotation; given this, and the fact that residents do not initiate their asynchronous curriculum until the PGY-1 rotation, the decision was made to not evaluate or include scans performed prior to the PGY-1 rotation.

**Population Health Research Capsule**

What do we already know about this issue?

*There is variability in how EM residents learn point-of-care ultrasound, with some studies demonstrating a decline in skills as they progress through training.*

What was the research question?

*We evaluated trends in ultrasound skills longitudinally at various points in a resident’s training.*

What was the major finding of the study?

*Over time, we found no differences in accuracy (97.1%) or acceptability (82.9%) of scans, or proportion of technically limited studies (14.5%, P >0.05 for all).*

How does this improve population health?

*Understanding nuances to training healthcare professionals in ultrasound can build capacity in local health systems and in resource-limited settings.*

Two independent reviewers (MF, MV) were trained to abstract data from QPathE using a standardized protocol taught by the study principal investigator (HK) in a two-hour training period. To ensure quality, a random sampling of 50 single cases from the entire cohort was collected by the principal investigator and cross-checked with the pull by the independent reviewers. We calculated overall agreement for the five above variables (total number of scans obtained, accuracy of interpretation, acceptability for credentialing, and number of TLS studies) to be 100% for each individual variable.

### Inclusion and Exclusion Criteria

All EM residents were included if they completed a dedicated, four-week PGY-1 ultrasound rotation. Residents were excluded if they had interruptions in their rotation (ie, due to the COVID-19 pandemic), or interruptions in their residency training and/or were not available for analysis at the 24-month time mark. For evaluation of the trend over time, we excluded residents if they did not complete any additional POCUS exams in the 24-month time frame. The following exam types were included for evaluation: aorta; biliary; cardiac; focused assessment with sonography in trauma (FAST); renal; soft tissue/musculoskeletal (ST/MSK); and thoracic. We excluded bowel, deep venous thrombosis, early pregnancy, and ocular studies due to low overall numbers of these exams (attributed to institutional-specific factors).

### Internal Ultrasound Education/Training Requirements

During the study period, all EM residents completed a consecutive four-week rotation, shared with an anesthesiology rotation, at various time points in the PGY-1 year. All EM residents completed their rotation primarily at the academic medical center ED with ~65,000 annual visits per year. Residents were required to complete at least 100 POCUS studies by the end of the PGY-1 rotation, 25 of which were required to be FAST examinations; this requirement was initiated in 2019. At the time of residency graduation, residents were required to complete a total of 150 POCUS examinations. Residents had the option to schedule additional ultrasound elective experiences as individual ultrasound shifts, two-week rotations, four-week rotations, or split with an additional elective in the PGY-2 and/or PGY-3 years (15% of the study cohort completed at least one elective experience, although the individual experiences were not tracked). Additional details about the PGY-1 rotation are highlighted in the Supplementary Materials (Appendix 1a).

### Clinical Operations and Ultrasound QA

Ultrasound machines for clinical use varied during the study period. They included Edge I/II and M-Turbo (Fujifilm Sonosite, Inc, Bothel, WA) and Sparq ultrasound systems (Philips NV, Amsterdam, Netherlands), all with curvilinear, linear and phased array transducers. The PGY-1 resident on their rotation used a cart-based ultrasound machine, the Edge II, purchased solely for educational use, with three standard probes (5–2 MHz curvilinear probe, 5–1 MHz phased array probe, and 10–5 MHz linear probe). The rotation required dedicated scanning in the department for a minimum of 20 hours/week for the duration of the rotation. When scanning independently, all residents were required to scan patients with confirmatory imaging.

To earn credit, residents were required to obtain a specific number of views for each study indication, which was shared with the resident at the start of their PGY-1 rotation. (See Supplementary Materials, Appendix 1b) for the required views for ultrasound indications). The resident was required to fill out a standardized worksheet for the study in QPathE and submit the study for quality assurance (QA). Four ultrasound fellowship-trained faculty performed weekly QA on all POCUS examinations on a rotating schedule. During these sessions, the ultrasound-fellowship trained faculty member assigned a measure for the accuracy of the residents’ interpretation of the images (eg, true positive [TP], true negative [TN], false positive [FP], false negative [FN]), whether the images were considered to be TLS, and whether the scan was acceptable for credit ([Yes/No]). To have the exam accepted for credit, the resident 1) must have obtained the correct number of ultrasound images and 2) obtained images that were interpretable by the faculty member (ie, not TLS), and have made a correct interpretation of the clips (eg, TP or TN). Residents were not given partial credit for incomplete exams and were given credit only for exams they personally performed (ie, no shared or split credit for scans obtained with other residents or students). Only POCUS examinations that were submitted for QA were analyzed.

### Statistical Analysis

Total number and percentages were collected via analytics in QPathE. We evaluated three measurements of performance at each time point for accuracy, TLS, and acceptability percentages. We defined accuracy as the percentage of adequate scans that were correctly interpreted (TP + TN/TP + TN + FP + FN). The TLS was defined as what percentage of scans were inadequate for interpretation (TLS/TP + TN + FP + FN + TLS), and acceptability was defined as the percentage of scans that were approved for resident credit (TP+TN/TP+TN+FP+FN+TLS).^9^ The pathology percentage was also collected at the end of the PGY-1 rotation and at the end of the study period (24 months). Pathology was defined as the number/percentage of true positive and false negative studies over all scans that were adequate for interpretation (TP+FN/TP+TN+FN+FP).

We analyzed performance characteristics over the course of a resident’s training period using mixed-effects binomial logistic regression with time as a fixed-effect factor and treating academic year and subject (within year) as random factors. These models involve ratios of counts (ie, *y/m*) as the response computed separately for each resident. Analysis of TLS and acceptable scans both used *m* = TP+TN+FP+FN+TLS as the binomial denominator and *y* = TLS or TP+TN as the numerator for TLS and acceptable performance measures, respectively. Accuracy involved the number of correctly classified findings (*y*=TP+TN) relative to the total number of studies that were not technically limited (*m*=TP+FP+TN+FN). Models were fit separately for each performance characteristic, and likelihood ratio tests were performed to determine whether performance varied over time. We checked all models for adequacy via half-normal plots of the deviance residuals.^10^

## RESULTS

### Totals and Overall Accuracy, TLS, and Acceptability Percentages

In total, 70 residents met initial criteria for evaluation. Four residents were excluded (two due to interruptions in training and two due to interruptions in their PGY-1 rotation due to the COVID-19 pandemic). One additional resident was excluded due to completing no scans in the 24-month study period, leaving 65 total residents for the trend-over-time analysis. Of these residents, 20 (30.7%) were female and 45 (69.2%) were male. These 65 residents completed (with QA) a total of 13,229 POCUS examinations during the study period.

In the PGY-1 rotation, the most common study performed was the FAST examination, which accounted for 22.2% of all exams performed. Thoracic (20.5%) and renal (19.0%) studies were the next most common applications. At the end of the study period (24 months post-PGY-1 rotation), FAST remained the most commonly performed exam type (25.4%). Cardiac examinations were the second most common, accounting for 20.4% of all studies obtained at this time point. Overall accuracy for all included residents and exam types during the study period was 97.1% (95% CI 96.2–98.0%); TLS was 14.5% (95% CI 9.7–20.6%); and acceptability was 82.9% (95% CI 76.3–88.2%). These findings are summarized in Tables 1 and 2.

### Trends in Accuracy, Technical Limitations, and Acceptability % Between 3–24 Months Post-PGY-1 Rotation

**Figure 1:**
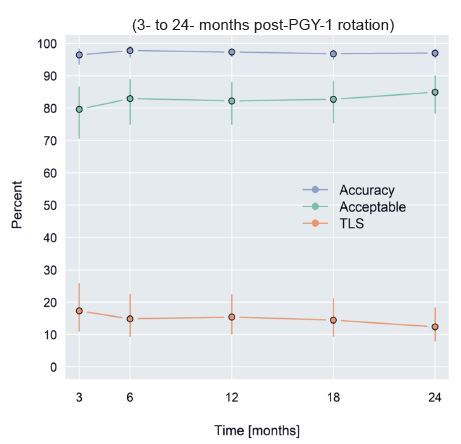
Trend of accuracy, technically limited studies, and acceptability percentages over time. 95% confidence intervals (CI) on the figure are noted as the whiskers extending from each plotting symbol. *TLS*, technically limited study.

Accuracy percentage during the study period (3–24 months post-PGY-1 rotation) was 97.1% (95% CI 96.2–98.0%) and did not vary over time (*P* = 0.84). The percentage of technically limited studies during the study period was 14.5% (95% CI 9.7–20.6%) and did not vary over time (*P* = 0.20). Acceptability percentage during the study period was 82.9% (95% CI 76.3–88.2) and did not vary over time (*P* = 0.28). Evaluation by individual exam types, analysis for any effect, as well as the trend between the five various time points, demonstrated no significant findings. These findings are summarized in Figure 1, Table 3, and Table 4.

## DISCUSSION

This study highlights trends in POCUS use, including number and types of exams performed, as well as the accuracy, TLS, and acceptability percentages in exams performed by EM residents at five time points beyond the dedicated PGY-1 rotation. Our data highlights several findings, including differences in use of POCUS in the PGY-1 rotation and in the 24 months post-rotation. Additionally, our data also highlights a stability and maintenance of POCUS image interpretability, acceptability, and technically limited studies for at least up to 24 months after a focused PGY-1 rotation.

Use of and experience with POCUS seemed to vary between the PGY-1 rotation and at the end of the study period. Only 14.4% of examinations performed in the PGY-1 rotation were cardiac exams, although at the end of the study period, cardiac exams accounted for 20.4% of all exams. Use of the FAST remained high both in the PGY-1 rotation (22.2%) and at the end of the study period (25.4%). This may be the result of various factors, including the requirements for scanning on patients with confirmatory imaging in the PGY-1 rotation, exposure to core EM faculty credentialed to perform various POCUS studies, and the overall training environment internal to our institution, with in-house 24/7 radiology technicians to complete confirmatory exams.

Aorta, renal, and thoracic studies declined in use between PGY-1 rotation and the end of the study period. It is likely that the types of scans performed in the PGY-1 rotation were influenced by the requirements of the rotation, while the data at the 24-month time point was likely more influenced by the scans that are deemed clinically useful in the ED environment, accounting for the difference observed at these later time points. These findings may be of value to ultrasound educators creating training POCUS curriculums.

Our study also highlights that POCUS skills (eg, accuracy of interpretation and acceptability for credit) are maintained up to at least 24 months post PGY-1 rotation. This is in contrast to the findings by Schleifer et al, who observed a decline in POCUS skills as residents progressed through training. This study also demonstrated that TLS rates increased from 4.7% to 13.6% from PGY-1 to PGY-4, while accuracy remained stable.^9^ Numerous differences in training experiences may account for this. For example, in the Schleifer study PGY-4 residents served in a supervisory role, and thus this decline could be attributed to less hands-on scanning in lieu of supervising junior residents. This is suggested by not only a drop in POCUS performance in the PGY-4 year, but also a decrease in the total number of scans obtained in later years. In our three-year training program, PGY-3 residents do not directly supervise junior residents. A multicenter analysis of comparative residency programs may be helpful.

Our trend-over-time analysis demonstrated that accuracy remained quite high (97.1%) when studies were deemed technically adequate. This may serve as a proxy for image *interpretation* skills. One theory as to why this may be is that residents may not have submitted exams for QA if they perceived them to be of suboptimal quality. However, upon closer evaluation of our data, we found that approximately 88% of scans where the resident was listed as the “operator” were ultimately submitted for QA. Another theory is that perhaps residents perform and submit mostly normal studies to obtain credit and reach their requirements for graduation. Evaluation of the percentage of pathology highlighted that most exams performed in the PGY-1 rotation were normal, which also may have contributed to the high accuracy. Additionally, the TLS percentage remained unchanged over the study period. Given limitations of QPathE and the volume of studies, we could not further elucidate why specific images were deemed technically limited. Further study of why studies are rejected would be helpful to know as potential targets for POCUS re-education or emphasis in later years of training.

Ultimately, this study highlighted several interesting differences between residents’ use of POCUS in the PGY-1 rotation and as they progressed through training in their day-to-day clinical shifts. Given the observed differences, there seems to be value and unique exposures in both experiences. In the PGY-1 rotation, residents focused on gaining skills and were exposed to largely normal examinations, while in their day-to-day practice as they progressed in training, residents focused on clinically impactful studies (such as cardiac and FAST exams) and had more exposure to pathology. Our findings also demonstrate that accuracy, acceptability, and the percentage of TLS did not significantly vary over time, suggesting that POCUS skills are maintained from their initial PGY-1 ultrasound rotation to each time point, up to 24 months post PGY-1 rotation examined in this study.

## LIMITATIONS

Our study does have several limitations. We included only scans submitted for QA; residents may been self-selecting which exams they submitted for credit, thus resulting in the high accuracy and acceptability that was observed in this study. Next, given that residents in training were required to have confirmatory imaging, their independent review of these results may have also influenced their completion of their QA worksheets in the PGY-1 rotation and potentially further influenced accuracy of interpretation and acceptability. In clinical practice, however, confirmatory imaging is not required if the EM attending supervising is credentialed to perform POCUS, and no differences in acceptability, accuracy, or TLS percentages were observed in the trend-over-time analysis. Next, given limitations of selections in QPathE and due to the high volume of scans, we could not evaluate individual cases to understand why an exam was deemed to be TLS or not acceptable. Given the high percentages of accuracy and acceptability that were observed, we hypothesize that this may have been due to technical issues surrounding image acquisition; however, future reviews in understanding rejected scans are needed.

Next, we did not adjust for timing of the PGY-1 rotation and exposures to POCUS prior to a resident’s PGY-1 rotation, and whether skills learned prior to the PGY-1 rotation could also have accounted for the observed high percentages of accuracy and acceptability. However, only 5% of residents’ total number of scans performed were obtained prior to the PGY-1 rotation, and most POCUS learning seems to start in the PGY-1 rotation. Many other factors may influence the use of POCUS and could not be adjusted for, including prior experience in POCUS in medical school curricula, exposure to a broader EM faculty group with varying credentials in POCUS, and exposure to specific pathology. A multicenter study evaluating residents’ use of POCUS would be helpful to the greater community for broader awareness on where to focus POCUS educational efforts.

## CONCLUSION

In this single-center study, residents’ use of and experience with point-of-care ultrasound varies between the PGY-1 rotation and post-PGY-1 rotation time periods. Accuracy, acceptability, and percentage of technically limited studies did not significantly vary over time, suggesting that learned POCUS skills in a PGY-1 rotation are maintained at these various timepoints, including up to 24 months post PGY-1 rotation. These findings appeared to be stable even when analyzed by individual exam types.

## Supplementary Information



## Figures and Tables

**Table 1:** Total number of ultrasounds by exam type and the percentages of scans that were accurate and acceptable or technically limited.

Exam	Total (%)	Accuracy % (95% CI)	TLS % (95% CI)	Acceptability % (95% CI)
*All Scans*	13,229	97.1 (96.2–98.0)	14.5 (9.7–20.6)	82.9 (76.3–88.2)
Aorta	960 (7.3)	100 (n/a)	16.5 (10.1–23.1)	81.9 (75.9–88.4)
Biliary	1,789 (13.5)	98.1 (95.3–99.6)	13.5 (9.5–19.0)	83.6 (77.4–89.0)
Cardiac	2,116 (16.0)	98.0 (96.0–99.3)	12.4 (7.4–19.1)	84.7 (77.5–90.4)
FAST	3,051 (23.1)	96.7 (95.0–98.4)	22.5 (14.5–32.0)	75.1 (65.3–83.7)
Renal	2,235 (16.9)	97.2 (94.0–99.4)	9.1 (4.2–18.7)	88.3 (76.8–95.9)
ST/MSK	638 (4.8)	97.9 (95.9–100.0)	9.3 (4.3–15.8)	88.4 (82.6–93.2)
Thoracic	2,240 (18.4)	95.9 (93.6–98.0)	13.1 (9.0–17.2)	83.8 (79.2–88.5)

*CI*, confidence interval; *FAST*, focused assessment with sonography in trauma; *ST/MSK*, soft tissue/musculoskeletal; *TLS*, technically limited study.

**Table 2:** Number and type of scans by ultrasound examination over 24-month period, and percentage of their accuracy, acceptability, and technical limititations.

	Aorta	Biliary	Cardiac	FAST	Renal	ST/MSK	Thoracic
PGY-1 Rotation							
Number of Scans, n(%)	761 (7.8)	1,288 (13.3)	1,400 (14.4)	2,159 (22.2)	1,846 (19.0)	271 (2.8)	1,990 (20.5)
Accuracy %	99.8	96.4	98.7	96.3	97.7	99.7	97.0
TLS %	27.5	22.7	17.5	24.3	16.7	10.0	14.1
Acceptable %	78.2	75.2	81.3	72.9	81.4	89.7	83.2
Pathology %	5.2	14.9	10.3	14.2	10.5	55.7	20.2
3 months post-rotation							
Number of Scans, n(%)	12 (3.9)	55 (17.8)	67 (21.7)	71 (23.0)	26 (8.4)	27 (8.7)	51 (16.5)
Accuracy %	100	94.9	98.2	95.8	80.0	100	97.2
TLS %	16.7	16.4	17.9	22.5	19.2	18.5	19.6
Acceptable %	83.3	80.0	80.6	74.6	69.2	81.5	76.5
6 months post-rotation							
Number of Scans, n(%)	27 (3.7)	110 (15.2)	140 (19.4)	185 (25.6)	81 (11.2)	84 (11.6)	96 (13.3)
Accuracy %	100	94.3	98.2	95.9	89.2	96.5	98.0
TLS %	25.9	16.4	13.6	20.5	16.0	15.4	14.6
Acceptable %	74.1	80.0	85.0	77.8	77.8	82.1	83.3
12 months post-rotation							
Number of Scans, n(%)	91 (5.5)	241 (14.4)	305 (18.3)	450 (27.0)	192 (11.5)	173 (10.4)	217 (13.0)
Accuracy %	100	95.6	98.1	94.9	92.3	97.9	97.8
TLS %	19.8	16.6	14.1	24.9	13.0	12.7	14.7
Acceptable %	80.2	80.1	83.9	72.7	82.8	86.1	82.5
18 months post-rotation							
Number of Scans, n(%)	133 (5.3)	361 (14.4)	478 (19.1)	678 (27.1)	278 (11.1)	261 (10.4)	314 (12.5)
Accuracy %	97.0	97.3	97.6	96.6	95.0	98.1	97.7
TLS %	18.0	16.9	15.1	26.1	12.2	10.7	13.7
Acceptable %	80.5	80.3	82.6	71.1	84.2	87.7	83.4
24 months post-rotation							
Number of Scans, n(%)	199 (5.7)	501 (14.3)	716 (20.4)	892 (25.4)	389 (11.1)	367 (10.4)	450 (12.8)
Accuracy %	96.7	97.4	96.8	97.1	96.3	97.5	96.8
TLS %	17.6	15.4	14.0	25.7	13.1	10.1	14.2
Acceptable %	80.9	81.8	83.5	71.9	83.8	88.0	82.2
Pathology %	15.2	22.9	25.5	18.1	27.5	70.9	36.8

Outcome measures were a cumulative measure of all scans performed to that specific time point.

*FAST*, focused assessment with sonography in trauma; *ST/MSK*, soft tissue/musculoskeletal; *TLS*, technically limited study.

**Table 3:** Accuracy, TLS, and acceptability % trend over time (3- to 24- months post-PGY-1 rotation).

Time	Accuracy % (95% CI)	TLS % (95% CI)	Acceptability % (95% CI)
***Overall***	**97.1 (96.2–98.0)**	**14.5 (9.7.20.6)**	**82.9 (76.3–88.2)**
+3 months	96.5 (93.6–98.3)	17.3 (11.1–25.7)	79.6 (70.7–86.5)
+6 months	97.8 (95.8–99.0)	14.9 (9.4–22.4)	83.0 (75.1–88.9)
+12 months	97.3 (95.9–98.4)	15.4 (10.1–22.3)	82.2 (75.0–88.0)
+18 months	96.7 (95.1–98.1)	14.5 (9.5–21.1)	82.7 (75.5–88.4)
+24 months	97.0 (95.5–98.2)	12.4 (8.0–12.8)	84.9 (78.5–89.9)
P-value	0.84	0.20	0.28

*TLS*, technically limited study; *CI*, confidence interval.

**Table 4:** Trends in accuracy, TLS, and accuracy percentages between 3–24 months post PGY-1 rotation by individual exam type with no observable differences noted. Estimates are based on mixed-effects binomial logistic regression. [a] = Likelihood ratio test for any difference in percentage among five time points (3, 6, 12, 18, and 24 months). [b] = Likelihood ratio test for whether percentages followed an increasing or decreasing trend over time.

Exam	Accuracy %	TLS %	Acceptable %
**Aorta**			
Number of residents, n	n=44	n=47	n=47
Average % (95% CI)	100	16.5 (10.1–23.1)	81.9 (75.9–88.4)
*P* (Any effect) [a]	N/A^1^	*P*=0.40	*P*=0.66
**Biliary**			
Number of residents, n	n=60	n=62	n=62
Average % (95% CI)	98.1 (95.3–99.6)	13.5 (9.5–19.0)	83.6 (77.4–89.0)
*P* (Any effect) [a]	*P*=0.88	*P*=0.72	*P*=0.69
**Cardiac**			
Number of residents, n	n=62	n=64	n=64
Average % (95% CI)	98.0 (96.0–99.3)	12.4 (7.4–19.1)	84.7 (77.5–90.4)
*P* (Any effect) [a]	*P*=0.86	*P*=0.23	*P*=0.32
*P* (Trend) [b]	*P*=0.31	*P*=0.21	*P*=0.50
**FAST**			
Number of residents, n	n=64	n=65	n=65
Average % (95% CI)	96.7 (95.0–98.4)	22.5 (14.5–32.0)	75.1 (65.3–83.7)
*P* (Any effect) [a]	*P*=0.31	*P*=0.55	*P*=0.27
*P* (Trend) [b]	*P*=0.95	*P*=0.43	*P*=0.45
**Renal**			
Number of residents, n	n=59	n=59	n=59
Average % (95% CI)	97.2 (94.0–99.4)	9.1 (4.2–18.7)	88.3 (76.8–95.9)
*P* (Any effect) [a]	*P*=0.29	*P*=0.60	*P*=0.26
*P* (Trend) [b]	*P*=0.07	*P*=0.92	*P*=0.35
**ST/MSK**			
Number of residents, n	n=63	n=63	n=63
Average % (95% CI)	97.9 (95.9–100.0)	9.3 (4.3–15.8)	88.4 (82.6–93.2)
*P* (Any effect) [a]	*P*=0.24	*P*=0.44	*P*=0.44
*P* (Trend) [b]	*P*=0.53	*P*=0.15	*P*=0.27
**Thoracic**			
Number of residents, n	n=56	n=58	n=58
Average % (95% CI)	95.9 (93.6–98.0)	13.1 (9.0–17.2)	83.8 (79.2–88.5)
*P* (Any effect) [a]	*P*=0.33	*P*=0.60	*P*=0.32
*P* (Trend) [b]	*P*=0.27	*P*=0.97	*P*=0.59

*FAST*, focused assessment with sonography in trauma; *ST*/*MSK*, soft tissue/musculoskeletal; *TLS*, technically limited scan.
