# Supplementary material for: Evaluation of Point-of-Care Ultrasound Use in Emergency Medicine Residents: An Observational Study
Source: West J Emerg Med. 2025 May 19;26(3):478–85. doi: 10.5811/westjem.21200 (PMC12208028; doi:10.5811/westjem.21200)
Supplement: Supplementary file 1 [file wjem-26-478-s001.docx]

**Appendix 1a.** *Internal Ultrasound Education/Training Requirements (continued)*

The educational curriculum included a structured program of asynchronous educational modules and quizzes on an online ultrasound education tool (Sonosim Inc., Santa Monica, CA, USA), which were required to complete by the end of the PGY-1 rotation. Residents were required to attend weekly ultrasound quality assurance (QA) sessions with an ultrasound-fellowship trained faculty member for feedback on image acquisition skills. All residents received dedicated, one-on-one orientation and scanning time with an ultrasound faculty member at least twice during their PGY-1 rotation. Lastly, ultrasound didactics and simulated experiences occurred at least six times per academic year as a part of residency conference. These sessions included a didactic delivered by an ultrasound-fellowship trained faculty member and various hands-on scanning experiences.

**Appendix 1b.** *Required Views for Ultrasound Indications*

***AORTA: 3 stills and 1 clip minimum***

- *Stills: transverse views WITH anterior-posterior measurements of: a) proximal aorta (with the SMA or Celiac in view), b) mid-abdominal aorta (after renal arteries), c) distal aorta (just above the aortic bifurcation)*
- *Clip: Sagittal view of aorta, sliding distal to ensure no saccular AAA. Clip must prove long axis view is aorta, either with visualization of celiac axis or IVC with aorta.*

***BILIARY: 2 clips and 1 still minimum***

- *Clips: scan through transverse and sagittal planes of gallbladder (with clear view of neck)*
- *Still: transverse gallbladder with measurement of anterior gallbladder wall*
- *Attempt measure common bile duct (optional)*

***CARDIAC:*** *One clip for cardiac standstill, otherwise* ***3+ clips minimum (3 of 4 cardiac views required):***

- *Parasternal long axis: demonstrating the RV, LV, mitral valve, aortic outflow tract, and descending aorta*
- *Parasternal short axis: demonstrating the RV and LV chambers, at the level of the papillary muscles*
- *Apical 4-chamber view: demonstrating at least 3 of 4 chambers (LV, RV, LA, RA)*
- *Subxiphoid: demonstrating at least 3 of 4 chambers (LV, RV, LA, RA)*
- *View of IVC in sagittal plane (optional)*

***Focused Assessment with Sonography in Trauma (FAST)****:* ***4+ clips minimum:***

- *Morrison’s pouch (coronal inferior liver tip, must be included)*
- *Left upper quadrant (coronal inferior diaphragm TO splenorenal TO paracolic gutter)*
- *Cardiac view (parasternal long or subxiphoid; demonstrating at least 3 chambers)*
- *Bladder (sagittal view)*

***RENAL: 5 clips minimum:***

- *Transverse and Coronal views of each kidney’s collecting system*
- *Bladder (clip preferred*
- *Bladder Volume (optional)*

***SOFT TISSUE: 4+ clips minimum:***

- *Transverse and Sagittal views area of interest*
- *One view of unaffected area/contralateral extremity. Must be labeled with body area*

***Musculoskeletal: Variable depending on what is being scanned:***

- *Knee: 4 clips minimum: sagittal quadriceps tendon, patellar tendon, medial and lateral joint lines*
- *Achilles: 2 clips minimum: sagittal and transverse clips of tendon*

***THORACIC: 4+ clips minimum:***

- *Anterior Chest: visualization of pleural line of each lung* OR M-Mode still of each lung*
- *Hemi-thorax: view of each hemithorax with coronal view of spine and diaphragm*

**If assessing for B lines: set appropriate depth and look >2 interspaces*
